# Using Recycled Tetrapak and Doped Titanyl/Vanadyl Phthalocyanine to Make Solid-State Devices

**DOI:** 10.3390/ma17020309

**Published:** 2024-01-08

**Authors:** María Elena Sánchez Vergara, Emiliano Toledo Dircio, Luis Alberto Cantera Cantera, Lourdes Bazán-Diaz, Roberto Salcedo

**Affiliations:** 1 Facultad de Ingeniería, Universidad Anáhuac México, Avenida Universidad Anáhuac 46, Col. Lomas Anáhuac, Huixquilucan 52786, Mexico; 2 Instituto Politécnico Nacional—ESIME, Unidad Profesional Adolfo López Mateos, Av. Luis Enrique Erro S/N, Gustavo A. Madero, Zacatenco, Ciudad de México 07738, Mexico; 3 Instituto de Investigaciones en Materiales, Universidad Nacional Autónoma de México, Circuito Exterior s/n. C.U., Ciudad de México 04510, Mexico

**Keywords:** Tetrapak electrode, semiconductor film, optical gap, solid-state device, electrical behavior

## Abstract

In this work we studied the semiconductor behavior of titanyl phthalocyanine (TiOPc) and vanadyl phthalocyanine (VOPc), doped with anthraflavic acid and deposited on Tetrapak/graphite as flexible electrodes. The molecular structure was approached using the density functional theory and astonishingly, it was found that the structure and electronic behavior can change depending on the metal in the phthalocyanine. Experimentally, the Root Mean Square was found to be 124 and 151 nm for the VOPc-Anthraflavine and TiOPc-Anthraflavine films, respectively, and the maximum stress was 8.58 MPa for the film with VOPc. The TiOPc-Anthraflavine film presents the smallest fundamental gap of 1.81 eV and 1.98 eV for indirect and direct transitions, respectively. Finally, the solid-state devices were fabricated, and the electrical properties were examined. The tests showed that the current–voltage curves of the devices on Tetrapak and VOPc-Anthraflavine on a rigid substrate exhibit the same current saturation behavior at 10 mA, which is achieved for different voltage values. Since the current–voltage curves of the TiOPc-Anthraflavine on a rigid substrate presents a defined diode model behavior, it was approximated by nonlinear least squares, and it has been determined that the threshold voltage of the sample for the different lighting conditions is between 0.6 and 0.8 volts.

## 1. Introduction

Organic electronic devices have seen rapid development with an emphasis on field-effect transistors [1], optoelectronic [2,3], photodetectors [4], memory devices [5] and gas chemosensors [6]. These devices are made up of organic semiconductors, which owe their behavior to specific properties in their structure, such as electronic delocalization, π–conjugated bonds, and in a significant number of these types of materials, the planarity of their molecular structure. Within these types of molecules, phthalocyanines stand out (Pcs), due to their multifunctional properties [7,8,9,10]. Some Pcs behave like semiconductors and exhibit photoelectric sensitivity; in other phthalocyanines, electrical conductivity can be induced by applying an electric field, and some show a photovoltaic effect. These characteristics have made them good candidates for different applications, such as the development of field-effect transistors [1], gas sensors [6], solar cells [11], and for the fabrication of photoswitches [12]. Besides, Pcs have unique physical properties, which allow them to act as dyes since they reflect light in the blue-green region of the visible spectrum. They can also act as photosensitizers, as they exhibit luminescence, fluorescence, and phosphorescence.

Pcs are organometallic compounds consisting of a ring containing four isoindole units linked by aza nitrogen atoms. The two central hydrogen atoms of the structure can be replaced by a wide variety of metal atoms, which can form covalent or ionic bonds with the macrocycle. The alkali and alkaline earth metals form ionic bonds with phthalocyanine. In contrast, other metals form covalent bonds with the two central nitrogen atoms and coordinate bonds with the remaining two nitrogen atoms. The most common metal phthalocyanines (MPcs) are those where the central metal ion has an oxidation state of 2+ (MPc). However, phthalocyanines with metal ions in oxidation states ranging from 3+ to 6+ are also known. According to the oxidation state of the metal, there are atoms or groups of atoms (X) ionically bonded, forming a (PcM)X_n_ structure. When X is a halogen covalently bonded to the aromatic ring, the structure (X_n_Pc)M is presented. Additionally, groups directly coordinated to the metal can exist, forming the structure (PcM)L_n_, where L is a Lewis base. In another case, oxidation of the central metal in MPcs can result in non-planar molecules like the vanadyl (VOPc) and titanyl (TiOPc) phthalocyanines [13]. These oxometal phthalocyanines are prone to two-dimensional π–π stacking, which leads to the presence of convex and concave pairs of these molecules in the crystal structure [14,15]. This results in a larger π–π stacking overlap, which renders promising high-performance organic thin film transistors based on these species [16,17,18,19]. However, the utilization of VOPc and TiOPc is rarely reported; these two phthalocyanines have not been extensively studied in optoelectronic or photovoltaic applications.

Besides, an important approach in modern organic electronics is the manufacture of optoelectronic devices, with components that can be recycled [20]. Regarding the above, the VOPc and TiOPc phthalocyanines have not been studied, although the interest in degradable electronics has been growing, with the purpose of reducing electronic waste, usually involving toxic materials that take long to decompose [21,22,23,24,25]. From this point of view, the materials used as a substrate play a major role, since the substrate supports the device and has direct contact with the electrodes. Among the degradable polymers that have been used as substrates are polyethylene terephthalate (PET) [26], polyvinyl alcohol (PVA) [27], poly lactic-co-glycolic acid (PLGA) [28] and naturally derived materials, such as cellulose [29,30]. In this sense, Tetrapak waste could be a good candidate because it is abundant and it is made of 71–75% cellulose, 5% aluminum and 20–24% polymer [20,31]. Besides, according to Platnieks et al. [31], Tetrapak is one of the most extensively used food packaging materials and a large quantity of this packaging ends up in landfills as garbage. Due to the above, the development of applications for Tetrapak waste is in demand, and recycling is a good option for reducing the disposal problem [32].

Therefore, in this work, we have studied the fabrication of flexible devices using recycled Tetrapak-based electrodes. For this purpose, Tetrapak juice containers were cut into pieces and a layer of graphite was deposited on those pieces to make them conductive. After this, we deposited on the graphite, a silver electrode composed of a layer of Tetrapak/carbon/silver (TCS). Finally, simple TCS/MOPc-Anthraflavine/Ag devices were fabricated, using anthraflavic acid-doped TiOPc and VOPc as the active layer. The main novelty of this work lies in the fact that, as far as we know, it is the first time that solid-state devices of this type have been manufactured. Furthermore, Tetrapak is proposed as a non-conventional substrate and electrode; this is a recycled, low-cost, light and flexible material. All these features are very important requirements for organic optoelectronic devices. In other context, the effect of a dopant such as anthraflavic acid on the semiconductor properties of TiOPc and VOPc is also studied. VOPc and TiOPc phthalocyanines have not been widely studied, and finally another novelty in this work is their study as organic semiconductors films.

## 2. Materials and Methods

### 2.1. TiOPc and VOPc Doping

**Figure 1:**
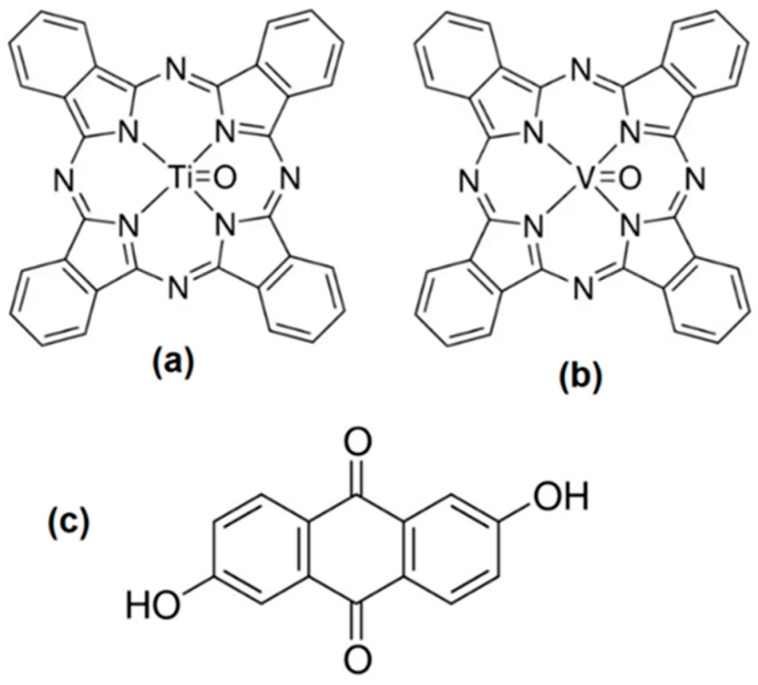
Structure of (**a**) titanyl phthalocyanine, (**b**) vanadyl phthalocyanine and (**c**) anthraflavine.

Oxytitanium phthalocyanine or titanyl phthalocyanine (TiOPc: C_32_H_16_N_8_OTi), vanadium(IV)phthalocyanine oxide or vanadyl phthalocyanine (VOPc: C_32_H_16_N_8_OV) and 2,6-dihydroxyanthraquinone or anthraflavic acid (anthraflavine; C_14_H_8_O_4_) (see Figure 1) were obtained from commercial sources (Sigma-Aldrich, Saint Louis, MO, USA) and were used without any further purification. Afterwards, a MOPc-Anthraflavine-doped semiconductor was obtained by dissolution of 200 mg (0.35 mmol) of MOPc and 100 mg (0.42 mmol) of anthraflavine in 6 mL of methanol. Doping was carried out for 30 min at 140 °C in a heated reactor Monowave 50 (Anton Paar México, S.A. de C.V. Hidalgo, México) with a pressure sensor. The reactor was operated with a borosilicate glass vial and manually closed by a cover with an integrated pressure (0–20 bar) and temperature sensor. The system was cooled and brought to atmospheric pressure, the MOPc-Anthraflavine was filtered, washed with methanol, and dried in a vacuum. To verify the main functional groups of the doped semiconductor, a FTIR spectroscopy analysis was performed, using a spectrometer Nicolet iS5-FT (Thermo Fisher Scientific Inc., Waltham, MA, USA) in KBr pellets. SEM micrographs were carried out in a JEOL 7600F Schottky Field Emission Scanning Electron Microscope with secondary electrons at 10 kV. The Energy Dispersive X-ray Spectroscopy (EDS) Oxford Inca-x act was used.

### 2.2. Fabrication of the Tetrapak/Carbon/Silver (TCS) Electrodes

The electrode was made with Tetrapak as follows: (i) the Tetrapak obtained from juice containers was washed with water, soap and ethanol several times, and after this, it was cut into pieces of 3 cm × 21 cm, (ii) the Tetrapak pieces were again washed with soap and methanol, and then dried for one hour at 100 °C. Each piece had a side completely covered by an aluminum + polyethylene film, and the other side was a surface of paperboard. (iii) The side covered by aluminum + polyethylene film was covered by a commercial graphitic carbon paint (the carbon paint was made by micro-graphite particles uniformly dispersed in isopropanol, and formulated with a small amount of a special polymer to give extra adhesive characteristics to the paint), with sheet resistance of 1.2 K ohms/sq. (iv) The perfectly painted pieces were left to air-dry at room temperature for one day and once the graphite was completely dried, conductive silver was deposited on the edge of the graphite layer and this would serve as current collectors. (v) Finally, the Tetrapak/carbon/silver (TCS) electrode was cut into pieces of 3 cm × 3 cm. Topographical and mechanical characteristics of the TCS electrodes were investigated with an atomic force microscope NaioAFM EasyScan 2, Naio (Nanosurf, Liestal, Switzerland), using an Ntegra with a measurement accuracy of 0.2 nm. The AFM was operated in static force contact mode, using the Stat0.2LauD silicon tip. The XRD analysis was performed with the θ–2θ technique using Bragg-Brentano Geometry with a diffractometer Bruker D8 Advance (Bruker Nano GmbH, Berlin, Germany) and working with CuK-α (λ = 0.15405 nm) radiation. The samples were measured at 0.6°/min, interval 5–70 and the grazing angle was 1.0°. The samples were also used to determine the optical absorption of the materials with a spectrophotometer Unicam UV300 (Thermo Fisher Scientific Inc., Waltham, MA, USA), in the wavelength range of 200–1100 nm.

### 2.3. Fabrication and Characterization of the Devices

The TCS electrode was coated by a film of MOPc-Anthraflavine semiconductor. For this, semiconductor films were deposited in a high vacuum deposition system Intercovamex (Intercovamex, S.A. de C.V., Cuernavaca, Morelos, Mexico), using tantalum crucibles, a vacuum pressure of 10^−5^ torr and a rate deposition of 1.4 and 6.3 Å/s for TiOPc-Anthraflavine and VOPc-Anthraflavine, respectively. Next, Ag was deposited on top of the semiconductor layer to act as a cathode, thus the configuration of the device was TCS/MOPc-Anthraflavine/Ag. Another reference device was also fabricated with a fluorine-doped tin-oxide (SnO_2_/F)-coated glass slide electrode also called FTO; the configuration in this case was glass/FTO/MOPc-Anthraflavine/Ag. The electrical behavior of the devices was procured through current–voltage (I–V) measurements in an auto-ranging pico-ammeter Keithley 4200-SCS-PK1 (Tektronix Inc., Beaverton, OR, USA). The samples were illuminated with commercial LEDs, using a lighting controller circuit from Next Robotix (Comercializadora KMox, S.A. de C.V., Mexico City, Mexico). I–V curves were obtained under darkness, white, red, orange, yellow, green, blue, and ultraviolet lights at room temperature from −1 to 1 V.

## 3. Results and Discussion

### 3.1. Chemical and Structural Characterization

**Figure 2:**
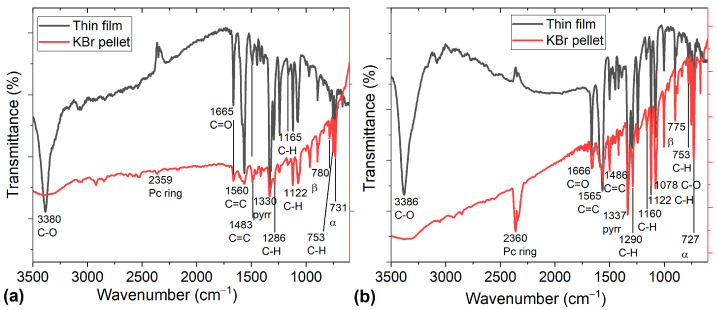
Infrared absorption spectra of (**a**) TiOPc-Anthraflavine and (**b**) VOPc-Anthraflavine in KBr pellets and thin film.

After carrying out the doping of the TiOPc and VOPc phthalocyanines with anthraflavic acid, IR spectroscopy was performed on KBr pellets, to corroborate the presence of the functional groups of both the phthalocyanines and the acid. After the fabrication of the MOPc-Anthraflavine thin films, their spectra were compared with those obtained in KBr pellets. This step was carried out to verify that the films did not suffer decomposition during their deposit. IR spectra of MOPc-Anthraflavine pellets and films are shown in Figure 2; these results indicate that the bands are in fact similar. The values of the representative vibrations of the MOPc-Anthraflavine are shown in the spectra: (i) the band responsible for the pyrrole in-plane stretch vibration in the Pc ring is observed in 1334 ± 3 cm^−1^, (ii) the bands located in 1288 ± 3, 1163 ± 3 and 1122 cm^−1^ are the result of the interaction between C of the peripheral rings, with the hydrogen atoms [33], (iii) the band located in 753 cm^−1^ is the interaction in the plane of C-H deformation, (iv) the band observed in 1563 ± 2 and 1485 ± 2 cm^−1^ results from a C = C stretching mode [33,34,35,36], (v) the signals in 729 ± 2 cm^−1^ and 778 ± 3 cm^−1^ stand for the α-phase and the β-phase, respectively, [34,37,38,39,40] and (vi) the signals of the anthraflavine in 3383 ± 3 and 1074 ± 4 cm^−1^ are for C-O and the signal in 1665 ± 1 is for C=O. The IR spectroscopy showed two important results: on one hand, when reviewing the spectra of the pellets, the presence of both phthalocyanine and anthraflavine is observed, which is an indication of a doping of both TiOPc-Anthraflavine and VOPc-Anthraflavine. On the other hand, when comparing the spectra of the pellet with its thin film, the same signals are observed. This is an indication that there was no decomposition or degradation by the MOPc-Anthraflavine semiconductors during the deposition process. These results are important because in the physical vapor deposition technique used to form the films, the MOPc-Anthraflavine is heated above its sublimation temperature creating a vapor, which then condenses on the substrate. According to Cranston et al. [38], it is expected that no vapor-phase chemical reactions occur so that the films are produced strictly through physical means. Conversely, during the chemical doping of the phthalocyanines, the purification of the obtained doped semiconductors was carried out to avoid the presence of impurities. Furthermore, the high vacuum evaporation technique used for the deposition of the material generates high purity films.

**Figure 3:**
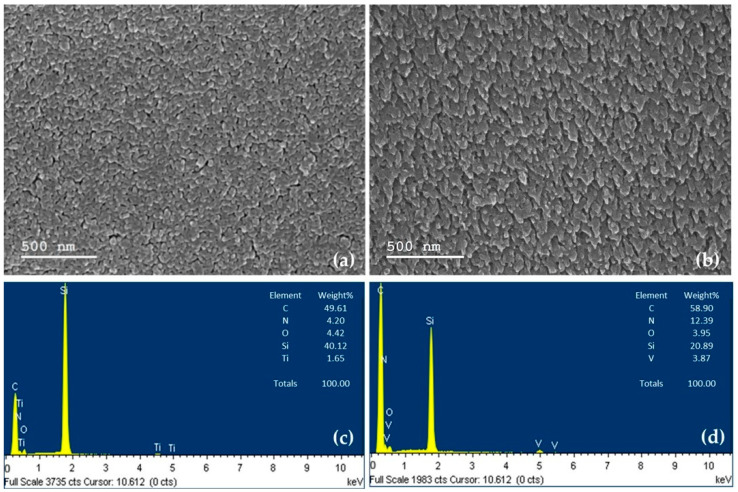
SEM images and EDS spectra of (**a**,**c**) TiOPc-Anthraflavine and (**b**,**d**) VOPc-Anthraflavine.

Figure 3 shows the SEM images as well as EDS spectra of both complexes. The morphology analysis of (a) TiOPc-Anthraflavine and (b) VOPc-Anthraflavine, reveals a pronounced high roughness in both cases. Notably, VOPc-Anthraflavine exhibits a larger grain size, representing the highest roughness and displaying a morphology reminiscent of stalactites, whereas TiOPc-Anthraflavine appears predominantly flat. Both films present a homogeneous morphology characteristic of the high vacuum evaporation technique. EDS analysis confirms the purity of the films with specific elements such as N, O, C and Ti or V for TiOPc-Anthraflavine and VOPc-Anthraflavine, respectively. The presence of silicon is due to the substrate.

**Figure 4:**
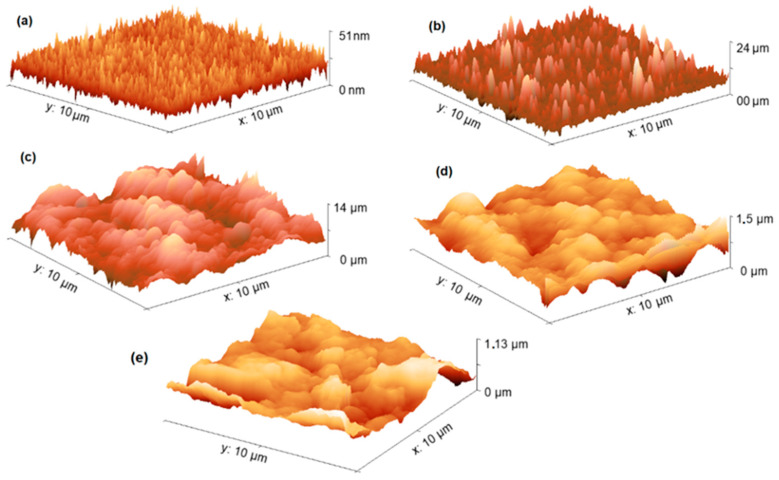
Three-dimensional AFM images of (**a**) TiOPc-Anthraflavine and (**b**) VOPc-Anthraflavine films on the n-silicon substrate and (**c**) TiOPc-Anthraflavine and (**d**) VOPc-Anthraflavine films on the (TCS) electrode. (**e**) TCS electrode.

Solid-state devices require to be integrated by homogeneous films of low roughness, in order to be applied in molecular electronics. The morphology of the MOPc-Anthraflavine films was analyzed by means of AFM. Figure 4 shows the 3D images for the TiOPc-Anthraflavine and VOPc-Anthraflavine films on the n-type silicon substrate and on the TCS electrode. This study was carried out with the purpose of comparing the morphology of films on flexible TCS, and films deposited on a rigid substrate. In phthalocyanines derivatives, two features of the substrate can have an important impact on nucleation and film growth, and they are physical surface roughness and surface chemistry. When comparing the topography of the films on silicon (Figure 4a,b), it is observed that both films are made up of an irregular surface with crests and valleys, formed by preferential directions of growth. These zones are thinner for the TiOPc-Anthraflavine film, whereas the VOPc-Anthraflavine film comprises larger structures, resulting in greater roughness. Table 1 shows the Roughness Average (Ra) and Root Mean Square (RMS) values for each film and shows that the VOPc-Anthraflavine film on silicon is practically twice as rough as the TiOPc-Anthraflavine film. The above is reflected in the different transport of electrical charges exhibited by the two films. Meanwhile, when comparing the films deposited on the TCS electrode (Figure 4c,d), a similar morphology is observed in both films, consisting of coarse grains. In this case, the lower roughness was observed in the VOPc-Anthraflavine film on the TCS electrode, although it should be noted that the films on this electrode exhibit much higher roughness than its titanium counterpart when they are deposited on silicon. This is an expected result because changing the substrate modifies the nucleation and growth process of the thin films, while the silicon used as a substrate has its surface polished, the TCS is made up of cellulose fibers that, as seen in Figure 4e, generate coarse grains and high roughness (Ra = 103.6 nm and RMS = 130.2 nm). Besides, MPcs with a nonplanar structure such as VOPc and TiOPc, are characterized by a change in their film’s morphology depending on the substrate material [41]. It was observed that MOPcs-Anthraflavine grew quicker on the silicon substrate which also reveals fewer defects on its surface, forming a greater number of nuclei, but with less growth of them. Furthermore, the high surface roughness in the TCS reduces the barrier for heterogeneous nucleation by decreasing the diffusion distance of molecules but with greater growth.

**Figure 5:**
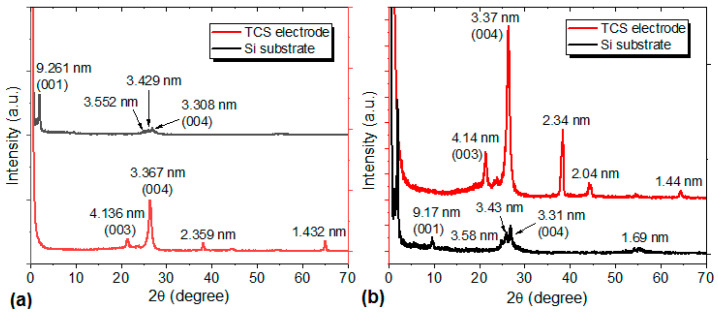
X-ray diffraction patterns on the silicon substrate and the TCS electrode of (**a**) TiOPc-Anthraflavine and (**b**) VOPc-Anthraflavine films.

In order to complement the previous information, X-ray diffraction was carried out on the films on the silicon substrate (Figure 5a) and the TCS electrode (Figure 5b). Physical surface characteristics and the intrinsic surface chemistry of the substrate can have an impact on film nucleation and growth [38]. Thus, a deep analyses of this technique was carried out, searching for answers on these topics. Important differences are observed in the diffractograms of Figure 5. Phthalocyanines have different polymorphs such as α and β [42,43], and these phases are categorized according to the crystalline stacking angle between the molecular plane and b-axis, yielding 65° and 45° for α and β, respectively [42,43]. The MOPc-Anthraflavine films on the silicon substrate exhibits a peak at 9.6°, related to the β-form in phthalocyanine [42] and the same peak is missing for the films on the TCS electrode, indicating that apparently, the structural organization of the β-form is disrupted [43]. However, the peak around 27° for all the substrates in Figure 5, corresponds to the β-form and indicates the growth of small crystallites lying parallel to the substrate surface plane and their stacking axes being inclined to it [44]. Besides, comparing the XRD results obtained for other VOPC-based films [45,46], the diffractograms in Figure 5b present very few peaks; however, in the 2θ region between 20° and 30°, the peaks reported in the literature remain. The differences are related to the used substrate, the deposition mechanism, the type of substituent and the metallic atom in the phthalocyanine. The small peaks in the diffractograms are associated with the low crystallinity of the films, although the crystallinity of the films on TCS electrodes is higher than that on silicon. Finally, and according to the results reported in the bibliography [20,47,48], the XRD patterns of the films deposited on TCS electrodes (see Figure 5b), depicts the band at 38.4°, which are associated with cellulose type I and type III [47,48]. While the peaks at 44.6 and 65.04° are attributed to the graphitic carbon randomly oriented on the Tetrapak surface [32]. It is necessary to highlight that the films on the TCS electrode have greater crystallinity compared to those deposited on silicon as was previously indicated. The TCS surface and its high roughness shows preferential sites for the heterogeneous nucleation and the growth of structures with crystallinity. This greater crystallinity is typically desired for many solid-state applications.

### 3.2. Mechanical and Optical Characterization

Regarding the mechanical properties of the films obtained by AFM, Table 1 shows how the maximum stress (σ) does not demonstrate significant variations between the films. These results indicate that the substrate does not exhibit a significant influence on the maximum stress that MOPc-Anthraflavine films can withstand. However, the type of substrate determines Knoop hardness (HK); in this case, the highest values are obtained for the films deposited on the TCS electrode. It is important to note that the HK values obtained are lower than the hardness for polymeric materials, which have an HK of around 22. However, the use of TCS electrodes doubles the hardness compared to rigid silicon substrates, which can be favorable for their use in electronic devices.

**Figure 6:**
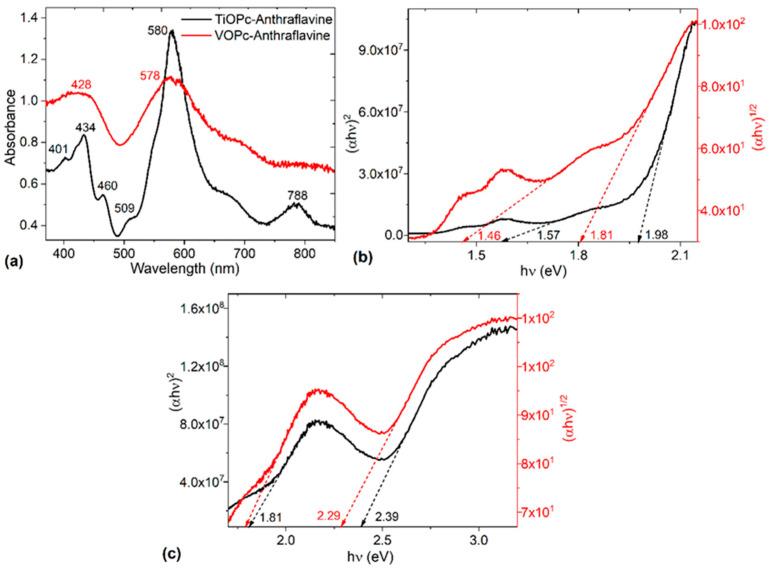
(**a**) Absorbance and Tauc plots for direct transitions (αhν)^2^ and for indirect transitions (αhν)^1/2^ of (**b**) TiOPc-Anthraflavine and (**c**) VOPc-Anthraflavine films.

In other context, and with the purpose of evaluating the optical behavior of the MOPc-Anthraflavine films, UV-vis spectroscopy was carried out. Figure 6a shows the absorbance spectra of the films. It is important to consider that these spectra are determined by the molecular orbitals within the aromatic π–electrons system and the overlapping orbitals on the central metal in the phthalocyanine [49]. In TiOPc and VOPc, strong absorptions are observed in the visible region between 570 and 720 nm (Q-band) and in the UV region between 370 and 460 nm (B-band) [14,50,51,52,53,54]. The Q-band representing the π–π* transition [49] originates primarily from electronic transitions from the highest occupied molecular orbital (HOMO) to the lowest unoccupied molecular orbital (LUMO) in the phthalocyanine core [54]. The molecular organization in the films affects the wavelength of the Q-band [50,55], hence the maxima of the Q-band in the spectra of TiOPc-anthraflavine and VOPc-anthraflavine films is located at 578 and 580 nm, respectively. Although the values are very similar, the spectrum of the TiOPc-anthraflavine film presents the second component or the shoulder shifted towards blue, which according to Bonegardt et al. [54], is a sign that in this film, the formation of aggregates with a cofacial arrangement of Pc macrocycles is presented. This shoulder at 509 nm in the TiOPc-Anthraflavine film is associated with the presence of an aggregated species [14,56]. Alternatively, the B-band is assigned to the transition between π–π* (b2u to eg) orbitals [49] and displays two peaks in the TiOPc-Anthraflavine spectrum and one broad peak as seen in the VOPc-Anthraflavine spectrum [38]. According to Cranston et al. [38], in the region of the B-band, the changes in the absorption spectra between the two films are thought to be originated by orbital overlap of the TiOPc and VOPc rings and the central metal. Additionally, the presence of the peaks in the TiOPc-Anthraflavine spectrum in the B-band region suggests the existence of π–d transitions as a result of the partially occupied d-orbitals of the titanium atom [38]. Finally, in the spectrum of the film with TiOPc, a band appears at 788 nm, referring to the charge transport between phthalocyanine and anthraflavic acid [52].

The Tauc method was used to calculate the direct and indirect energy band gap values from the UV-Vis spectra. The energy band gap (Eg) was obtained by analyzing the variation of the absorption coefficient (α) with the photon energy (hν) in the high absorption region as follows [50,57]:

(1) $$\alpha hv=A{\left(hv-E_{g}\right)}^{m}$$

where A is a constant and m is an exponent parameter that depends on the type of transition [50,57], m = 1/2 for indirect and m = 2 for direct forbidden electronic transitions. The relation between (αhν)^m^ vs. hν was plotted in Figure 6b,c to extract the values of the E_g_ by extrapolating the straight line portion. These figures and Table 2 present the values of the fundamental and the onset gaps for indirect and direct electronic transitions. The first portion of the Tauc plots can be referred to the energy onset gap and is defined as the formation of a bound electron–hole pair in the semiconductor’s materials, or Frenkel exciton [50,57]. The second portion could be attributed to the fundamental energy band gap between the HOMO and the LUMO [50,57]. The obtained values of these band gaps corroborated the calculated theoretical value. The TiOPc-Anthraflavine film is the one with the lowest band gap values, both for direct and indirect transitions. This may be due to the closed shell nature of Ti^4+^, which reduces the spin–orbital interactions between the Ti^4+^ and the π–electrons system of the Pcs. It is important to consider that the fundamental band gap for indirect transitions obtained in this study is lower than that obtained by some authors [58] for pristine TiOPc films (2.85 eV), and for the film-based planar heterojunction: poly (3,4-ethylenedioxythiophene) polystyrene sulfonate/titanyl phthalocyanine (2.91 eV). These fundamental band gap values for direct transitions are also interesting because they are slightly larger than the one obtained by Bandas et al. [59] of 1.75 eV for hybrid electrodes based on a Ti/TiO2 mesoporous/reduced graphene oxide which can be considered inorganic nature electrodes. Electrodes of organic nature, such as those presented in this work, usually have a much higher band gap than inorganic ones. The above is evidence of the positive effect of anthraflavic acid as a dopant. Additionally, there is no appreciable difference between the fundamental gaps obtained for direct transitions with respect to indirect ones. This reinforces the fact that the doping acid is the one that exerts the greatest influence on charge transport, with respect to the structure of the films and the type of electronic transition (direct for crystalline structure and indirect for amorphous structure).

### 3.3. DFT Analysis

Quantum chemical calculations can help to interpret the last optical parameters obtained. The geometry optimization of MOPc-Anthraflavine was carried out at the level of density functional theory (DFT) using the Gaussian package [60]. The molecular structure of all the involved species was optimized using the B3PW91 method [61,62] included in the Gaussian 16 pack [60], and the calculations were carried out using the basis set 6–31g**. Stable minimum energy configuration of each molecule was approached by calculating their frequencies.

Anthraflavic acid used in this study as a dopant, is a very versatile species [63], which bears several terminal groups with different capabilities of interaction with other molecules, groups or atoms. These groups can establish hydrogen bonds, and both electrostatic and covalent interactions, therefore they can participate in chemical processes of different nature. In particular its terminal aromatic rings resemble that of the phenol molecule, and it is known that phenol has acid behavior because the hydrogen atom joint to the oxygen in the hydroxyl group has a relatively weak bond, due to the aromaticity. It is said the aromatic current in the center of the ring compels the electrons of the oxygen to participate in the delocalization motion, and the consequence is that the hydrogen atom can be separated as a positive ion yielding an acid environment. Therefore, anthraflavic acid shows a similar behavior as phenol; it can lose the hydrogen atoms of its ends as positive ions and manifest the acid behavior. In the present case, the interaction between this compound and the metallic phthalocyanines can take two different paths. First, the ionized species can interact with the central metallic atom of the phthalocyanine group via the ionized terminal oxygen or can follow the hydrogen bond route with the interaction of the oxygen joint to the metal atom with the terminal hydrogen atoms of the anthraflavic acid species forming a classical hydrogen bond. The curious situation is that both possibilities are found in the present case because the first one is followed by the vanadium complex whereas the second one is the case of the titanium complex and in this sense none of the structures has a net charge when the interaction form the definitive complexes. Both cases will be analyzed for their part.

**Figure 7:**
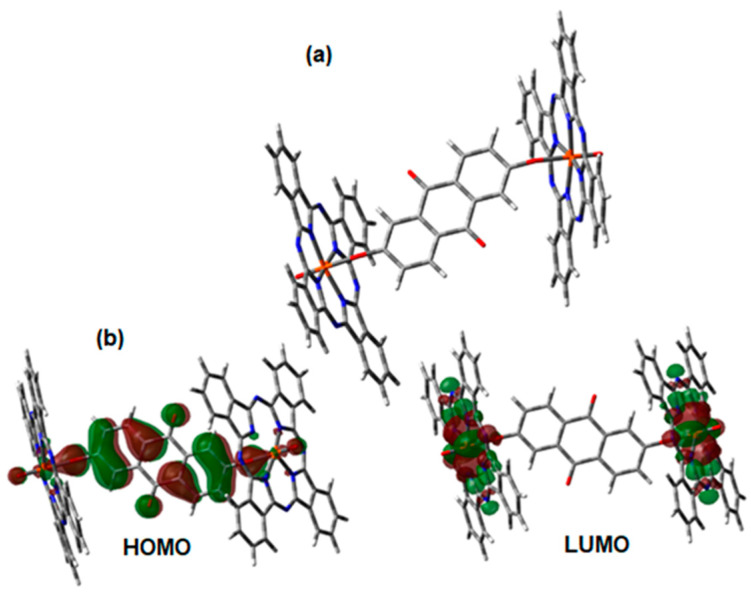
(**a**) Optimized structure of VOPc-Anthraflavine. (**b**) Frontier molecular orbitals of the VOPc-Anthraflavine.

The structure of VOPc-Anthraflavine is shown in Figure 7a and two phthalocyanine molecules interact with a central anthraflavic unit. The bond V-O is 1.95 Å at both ends whereas the Wiberg index for this same bond is 0.72 on average. This value confirms the presence of a moderate coordination covalent bond, the phthalocyanine moiety experiments a little deformation, when the planar square adopts a little ruffle-like shape with a deviation angle of 15.5°. The HOMO and LUMO of these species are shown in Figure 7b and as can be seen, the HOMO is located in the anthraflavic acid molecule and the LUMO is located in the phthalocyanine. From the above it is deduced that in thin films of this semiconductor, applied to electronic devices, the electrons would flow from the acid towards the VOPc.

**Figure 8:**
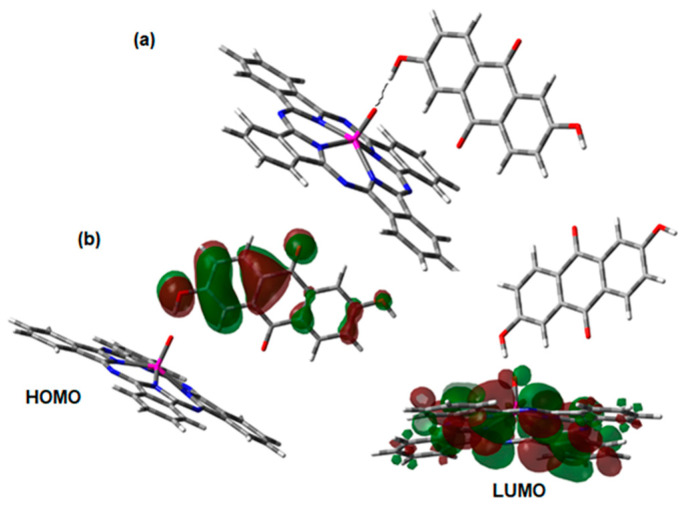
(**a**) Hydrogen bond between the complex of phthalocyanine of titanium and anthraflavic acid without ionization. The dotted line signals the hydrogen bond. (**b**) Frontier molecular orbitals of the TiOPc-Anthraflavine.

After the successful analysis of the vanadium complex, the analogue of titanium was similarly studied at the beginning. However, the new structure is an ionized specie, and this feature always generates perturbation in the ordering of molecular orbitals and indeed the calculations were more difficult than those performed for vanadium, therefore another possibility was considered. Hydrogen bonds have been studied by our group in other semiconductor behavior cases [64]; therefore, the next study is based on the probability that the titanium complex can form one interaction of this kind with the anthraflavic acid molecule. The hydrogen bridge is localized between the terminal hydrogen atom of the acid and the apical oxygen atom of the phthalocyanine. The shape of the molecule is shown in Figure 8. The length between the hydrogen and oxygen atoms is 1.82 Å, the Wiberg index is 0.109 and the energy contribution found taking advantage of the Grimme module application is 7.1 kcal/mol; all this data demonstrate it is a strong dispersion interaction. Making a comparison between this configuration and that which is similar to the one found for the vanadium complex by means of the Boltzmann distribution analysis, the case of the hydrogen bond is favored by 5.5 kcal/mol, and the specie remains neutral. The HOMO and LUMO of TiOPc-Anthraflavine are shown in Figure 8b, and an overview of the results is shown in Table 3. In the same way as that obtained for VOPc-Anthraflavine, the HOMO is located in the acid and the LUMO in the structure of the phthalocyanine, so the flow of electrons in the doped semiconductor would be towards this molecule, through the bridge of hydrogen.

Table 3 presents the gap values obtained through theoretical calculations, and when comparing them with the optical gaps experimentally obtained (see Table 2), it is observed that for the VOPc-Anthraflavine species, the theoretical value is lower. This is because DFT calculations consider isolated molecules without any type of interaction. Regarding the TiOPc-Anthraflavine species, the opposite behavior was observed: the theoretical gap is larger than that experimentally obtained. This is because the proton of anthraflavic acid is practically “loose”, interacting with both the oxygen of anthraflavic acid and the titanium atom to establish a “banana” bond [65]. This bond has three atoms and a single electron pair, so oxygen and titanium are pseudo charged and there is ionic density in this vicinity. Finally, it is necessary to mention that, although there are differences between the values of the optical gaps and the theoretical gaps, both are in the same order of magnitude, and are also in the range of organic semiconductors.

### 3.4. Electrical Characterization

**Figure 9:**
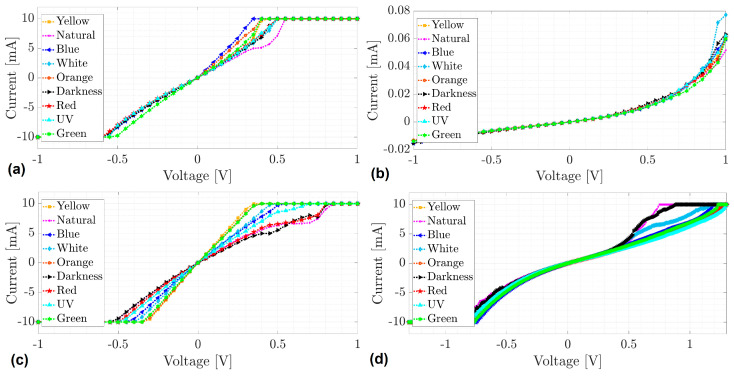
I–V curves from (**a**,**c**) TCS/MOPc-Anthraflavine/Ag and (**b**,**d**) FTO/MOPc-Anthraflavine/Ag devices under different lighting conditions.

In order to evaluate the electrical behavior of the MOPc-Anthraflavine films, simple solid-state devices were manufactured, using TCS electrodes. The TCS/MOPc-Anthraflavine/Ag flexible devices were fabricated using anthraflavic acid-doped TiOPc and VOPc as the active layer. The electrical behavior obtained for each flexible device was compared with that obtained for rigid devices manufactured on the FTO-coated glass slide: FTO/MOPc-Anthraflavine/Ag. The results are presented in the current–voltage (I–V) graphs of Figure 9 under different lighting conditions to detect photoconductive properties of the samples. It is important to consider that Ag, which acts as cathode in all devices, has a work function (Φ) of 4.2 eV, and is the electrode that provides electrons to the semiconductor through its LUMO. Based on its Φ and the LUMO value reported in Table 3, the electron transit to the device is expected to be more efficient with VOPc-Anthraflavine. In graphs I–V this behavior is actually observed, and even the device on FTO (Figure 9d) is the one with the highest transported current. This may be due to the lower energy barrier between the silver cathode and the LUMO of the VOPc-Anthraflavine semiconductor, but also due to the lower energy gap between the HOMO and the LUMO of this same species.

From Figure 9, the I–V curves of the samples TiOPc-Anthraflavine on TCS (Figure 9a), VOPc-Anthraflavine on TCS (Figure 9c) and VOPc-Anthraflavine on FTO (Figure 9d) present a saturation current of 10 mA. The samples have the same conduction behavior, regardless of the type of material used and it can also be seen that the saturation current is reached at different voltages for each type of light. Table 4 shows the voltage at which the saturation current is reached for the different lighting conditions.

As already mentioned, samples TiOPc-Anthraflavine on TCS, VOPc-Anthraflavine on TCS and VOPc-Anthraflavine on FTO present a saturation current of 10 mA regardless of the material used; however, as can be seen in Table 4, each sample reaches saturation at different values of the applied voltage, which also changes for different lighting conditions. Specifically, the sample TiOPc-Anthraflavine on TCS saturates at lower voltage levels than samples VOPc-Anthraflavine on TCS and VOPc-Anthraflavine on n-silicon, which means it has lower resistance to current flow. In contrast, sample VOPc-Anthraflavine on n-silicon saturates at higher voltage levels compared to devices on TCS, which means it has higher resistance to current flow. In this sense, it is well known that the generated currents due to edge effects, may be important contributions to the total current in semiconductors, giving misleading results and causing variations of current [66,67,68]. However, it has also been reported that for semiconductors with lower resistivity, the edge effect plays a minor role on normal surfaces [68]. Since the samples have an average static resistance of less than 100 ohms, the currents generated by the edge effect will be neglected.

At another context, the I–V curve of the sample TiOPc-Anthraflavine on FTO (Figure 9b) does not present a saturation behavior in the voltage range used. Since its behavior is closer to the characteristic curve of a diode, the following presents the approximation of the I–V curves of the sample TiOPc-Anthraflavine on FTO by the Shockley diode equation [69] defined by Equation (2).

(2) $$i\left(v\right)\;=\;I_{s}\left(\;\exp\left(\frac{1}{nV_{T}}v\right)-1\right)$$

From Equation (2), $i\left(v\right)$ is the current through the diode, $I_{s}$ is the saturation current, v is the applied voltage, n is the emission coefficient of the device and $V_{T}$ is the voltage due to temperature. Since the parameters $I_{s}$, n and $V_{T}$ are unknown for the sample TiOPc-Anthraflavine on FTO, they were estimated using the I–V data from Figure 9b and applying the nonlinear least squares method [70] to Equation (3).

(3) $$i\left(v\right)\;=\;I_{s}\left(\;\exp\left(av\right)-1\right)$$

Since $a=\frac{1}{nV_{T}}$, the parameter estimation of Equation (3) consists of determining the parameters $I_{s}$ and a that fit the equation to the I–V data. Table 5 shows the estimated parameters of the Shockley diode Equation (3) for the different lighting conditions in Figure 9b of the sample TiOPc-Anthraflavine on n-silicon.

**Figure 10:**
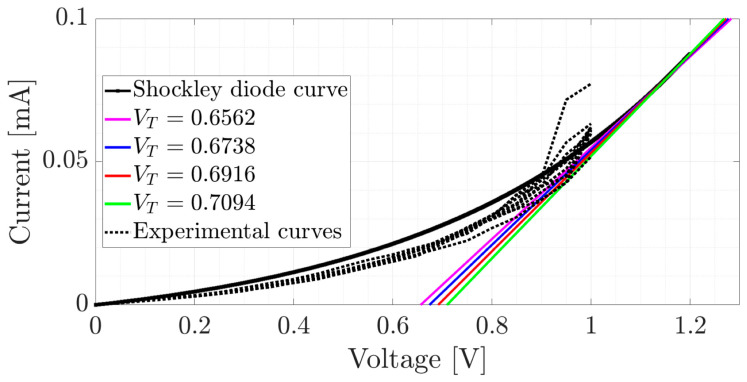
Approximation of the threshold voltage $V_{T}$ of the device, $I_{s}=0.01$, a=1.9.

The results in Table 5 show that the estimate of the saturation current $I_{s}$ in all cases approaches 0.01, while the parameter $a=\frac{1}{nV_{T}}$ changes for each type of lighting and on average is equal to 1.9. Besides, the threshold voltage of a diode denoted by $V_{T}$ is the minimum voltage necessary to reach the conduction zone of the diode [69]. The threshold voltage can be determined graphically, by fitting a straight line in the conduction zone of the diode and the intersection of the straight line with the voltage axis which represents the threshold voltage $V_{T}$. A linear approximation at $v^{*}$ of the Shockley diode Equation (3) is the tangent straight line at $v^{*}$ to the curve (2) given by Equation (4):

(4) $$y=i^{\prime }\left(v^{*}\right)x+\left(i\left(v^{*}\right)-i^{\prime }\left(v^{*}\right)v^{*}\right)$$

where $i^{\prime }\left(v^{*}\right)$ denotes the first derivate of Equation (2) evaluated in $v^{*}$. Based on the Shockley diode Equation (2) and using the tangent line (3) at different points $v^{*}$ as the straight line in the conduction zone of the diode, Figure 10 shows approximations of the threshold voltage $V_{T}$ of the device.

Based on the approximations of the Shockley diode equation and the tangent straight lines in Figure 10, the threshold voltage of sample TiOPc-Anthraflavine on FTO changes for different lighting conditions. In this sense, for the voltage range used and the different types of lighting, it can be established that the threshold voltage of the sample can vary between 0.6 and 0.8 volts. From the study on the electrical behavior of the MOPc-Anthraflavine semiconductor films, it is observed that it is the devices with the VOPc-Anthraflavine film that present the greatest transport of electrical charges. However, the substrate influences charge transport more significantly in the device with the TiOPc-Anthraflavine film, although the use of recycled Tetrapak electrodes in both films is definitely a viable option in device manufacturing.

## 4. Conclusions

We have demonstrated that Tetrapak waste can be used to produce a novel class of flexible electrodes. For this purpose, semiconductor films of MOPcs of vanadium and titanium, doped with anthraflavic acid, were deposited on the electrodes. The IR study confirmed the chemical structure of the semiconductor films and their non-degradation when deposited on the electrodes. The topography and structure of the films show a high RMS above 120 nm; however, they also present an acceptable crystallinity, dominated by the β-phase of phthalocyanines. Regarding its mechanical parameters, the maximum stress of the films was obtained between 7.91 and 8.58 MPa and the hardness was 11.3 HK. Regarding the optical behavior of the films, the fundamental gap energy obtained from UV-vis spectral data are found to be between 1.81 and 2.29 eV and 1.98 and 2.39 eV, for indirect and direct electronic transitions, respectively. These values are in the range of the organic semiconductors and the DFT results help to support this behavior although they reveal that the interaction between the anthraflavic acid and the metallic phthalocyanine complexes can follow two different chemical pathways in which the neutral nature of the resultant cluster always remains. The first one occurs in the case of the vanadium complex and is represented by a deprotonated form of the anthraflavic acid which makes a direct bond with the metal atom. In other context, the titanium complex arises from the formation of a strong hydrogen bond between the coordinated oxygen atom of the phthalocyanine moiety and the terminal hydrogen atom of the anthraflavic acid; in both cases, the theoretical band gap result relatively resembles the experimental one and the electric charge carriers in the device can be considered to be electrons. On the other hand, electrical experiments reveal that there are changes in the conductivity of the devices and that they do not have photoconductive properties, because the current flow does not increase under different types of light; however, when the samples TiOPc-Anthraflavine on TCS (Figure 9a), VOPc-Anthraflavine on TCS (Figure 9c) and VOPc-Anthraflavine on FTO (Figure 9d) are exposed to different types of light, the saturation current at 10 mA is reached at different voltage values. Regarding sample TiOPc-Anthraflavine on FTO, it does not have the same saturation behavior as the previous ones but shows acceptable conductive properties with a threshold voltage between 0.6 and 0.8 V and less variation in the behavior of the I–V curves when exposed to different types of light. In general, the results of this work demonstrate that low-cost, efficient and flexible electrodes can be fabricated using recycled Tetrapak, and also that these electrodes are excellent for optoelectronic devices performance. It is important to mention that edge effects were not considered in this work and an interesting future work could reveal the existence of currents due to the edge effect, and thus, the projected task would be the experimentation of the same samples using guard-rings which avoid edge effects.

## Figures and Tables

**Table 1:** Roughness, thickness and mechanical parameters of MOPc-Anthraflavine films.

Sample	Ra * (nm)	RMS * (nm)	Thickness * (μm)	σ (MPa)	HK
TiOPc-Anthraflavine on silicon-n	4.122	5.229	6.5	8.81	4.81
VOPc-Anthraflavine on silicon-n	10.21	16.27	6.1	8.42	5.01
TiOPc-Anthraflavine on TCS	121.1	151.1	6.2	7.91	11.31
VOPc-Anthraflavine on TCS	99.3	124.2	6.7	8.58	11.34

* Measurement accuracy: 0.2 nm.

**Table 2:** Fundamental and onset gaps for indirect and direct electronic transitions.

Sample	Fundamental Gap for Direct Transitions (eV)	Fundamental Gap for Indirect Transitions (eV)	Onset Gap for Direct Transitions (eV)	Onset Gap for Indirect Transitions (eV)
TiOPc-Anthraflavine	1.98	1.81	1.57	1.46
VOPc-Anthraflavine	2.39	2.29	1.81	1.80

**Table 3:** HOMO, LUMO and theorical gap of the MOPc-Anthraflavine structures.

Doped Semiconductor	HOMO (eV)	LUMO (eV)	GAP (eV)
VOPc-Anthraflavine	−5.52	−4.22	1.3
TiOPc-Anthraflavine	−5.41	−3.21	2.2

**Table 4:** Activation voltage of the saturation current under different lighting conditions.

Light	Sample		
	VOPc-Anthraflavine on FTO	TiOPc-Anthraflavine on TCS	VOPc-Anthraflavine on TCS
Natural	0.75 V	0.55 V	0.85 V
Darkness	0.88 V	0.5 V	0.85 V
White	1.17 V	0.5 V	0.5 V
UV	1.3 V	0.5 V	0.7 V
Blue	1.2 V	0.35 V	0.55 V
Green	1.23 V	0.4 V	0.4 V
Yellow	1.25 V	0.45 V	0.35 V
Orange	1.26 V	0.4 V	0.4 V
Red	1.29 V	0.5 V	0.8 V

**Table 5:** Parameters of the Shockley diode equation under different lighting conditions.

Light	TiOPc-Anthraflavine on FTO	
	a	$I_{s}$
Natural	1.6193	0.0114
Darkness	1.7601	0.0114
White	2.8563	0.0042
UV	1.8700	0.0098
Blue	1.7473	0.0111
Green	1.6628	0.0109
Yellow	1.8589	0.0096
Orange	1.8055	0.0101
Red	1.7227	0.0109
Average	1.9	0.0099

## Data Availability

Data are contained within the article.
